# Uptake of Proactively Offered Online and Telephone Support Services Targeting Multiple Health Risk Behaviors Among Vocational Education Students: Process Evaluation of a Cluster Randomized Controlled Trial

**DOI:** 10.2196/19737

**Published:** 2021-01-06

**Authors:** Prince Atorkey, Christine Paul, Billie Bonevski, John Wiggers, Aimee Mitchell, Emma Byrnes, Christophe Lecathelinais, Flora Tzelepis

**Affiliations:** 1 School of Medicine and Public Health Faculty of Health and Medicine University of Newcastle Newcastle Australia; 2 Hunter New England Population Health Hunter New England Local Health District Newcastle Australia; 3 Hunter Medical Research Institute University of Newcastle Newcastle Australia; 4 Priority Research Centre for Health Behaviour Faculty of Health and Medicine University of Newcastle Newcastle Australia

**Keywords:** uptake, proactive offer, online support services, telephone support services, multiple health risk behaviors, vocational education students

## Abstract

**Background:**

A high proportion of vocational education students smoke tobacco, have inadequate nutrition (ie, low fruit and vegetable intake), drink alcohol at risky levels, or are physically inactive. The extent to which vocational education students will sign up for proactively offered online and telephone support services for multiple health risk behaviors is unknown.

**Objective:**

The aim of this study is to examine the uptake of proactively offered online and telephone support services for smoking, nutrition, alcohol consumption, and physical activity risk behaviors, individually and in combination, among vocational education students in the Technical and Further Education (TAFE) setting. The characteristics associated with the uptake of online or telephone services for smoking, nutrition, alcohol consumption, and physical activity risk behaviors were also examined.

**Methods:**

Vocational education students enrolled in a TAFE class in New South Wales, Australia, which ran for 6 months or more, were recruited to participate in a cluster randomized controlled trial from May 2018 to May 2019. In the intervention arm, participants who did not meet the Australian health guidelines for each of the smoking, nutrition, alcohol consumption, and physical activity risk behaviors were provided electronic feedback and proactively offered online and telephone support services. Uptake of support was measured by whether participants signed up for the online and telephone services they were offered.

**Results:**

Vocational education students (N=551; mean age 25.7 years, SD 11.1; 310/551, 56.3% male) were recruited into the intervention arm. Uptake of the proactive offer of either online or telephone services was 14.5% (59/406) for fruit and vegetables, 12.7% (29/228) for physical activity, 6.8% (13/191) for smoking, and 5.5% (18/327) for alcohol use. Uptake of any online or telephone service for at least two health behaviors was 5.8% (22/377). Participants who were employed (odds ratio [OR] 0.10, 95% CI 0.01-0.72) and reported not being anxious (OR 0.11, 95% CI 0.02-0.71) had smaller odds of signing up for online or telephone services for smoking, whereas participants who reported not being depressed had greater odds (OR 10.25, 95% CI 1.30-80.67). Participants who intended to change their physical activity in the next 30 days had greater odds (OR 4.01, 95% CI 1.33-12.07) of signing up for online or telephone services for physical activity. Employed participants had smaller odds (OR 0.18, 95% CI 0.06-0.56) of signing up for support services for at least two behaviors.

**Conclusions:**

Although the uptake of proactively offered online and telephone support services is low, these rates appear to be higher than the self-initiated use of some of these services in the general population. Scaling up the proactive offer of online and telephone services may produce beneficial health outcomes.

**Trial Registration:**

Australian New Zealand Clinical Trials Registry: ACTRN12618000723280; https://www.anzctr.org.au/Trial/Registration/TrialReview.aspx?id=375001.

## Introduction

### Vocational Education Settings and Multiple Health Risk Behaviors

Vocational education settings are mandated to offer education programs that equip students with technical qualifications that prepare them for specific occupations such as carpentry, hairdressing, plumbing, floristry, automotive engineering, and welding [1]. A high proportion of vocational education students compared with university students report tobacco smoking, poor nutrition, risky alcohol consumption, and physical inactivity [2,3]. For instance, in an Australian study, 35% of vocational education students reported smoking tobacco daily, 96% did not consume at least five serves of vegetables daily, 50% did not meet the recommended daily fruit consumption of at least two servings, 49% consumed alcohol at risky levels, 88% were physically inactive, and 98% engaged in multiple health risk behaviors [2]. It is important to target smoking, nutrition, alcohol consumption, and physical activity health risk behaviors because they are associated with an increased risk of chronic diseases and death [4]. The risk of chronic disease and mortality is higher when one engages in multiple smoking, nutrition, alcohol consumption, and physical activity risk behaviors [4,5]. Vocational education settings are predominately made up of young adults [2]; therefore, offering interventions that target these risk behaviors at an early age could reduce chronic diseases, mortality, and health care costs.

Smoking, nutrition, alcohol consumption, and physical activity health risk behaviors have been found to co-occur or cluster together in vocational education students [6-8]. For example, a study with community college students identified 3 clusters: *active, binge-drinkers with poor dietary intake*; *nonactive, moderate-smokers and nondrinkers with poor dietary intake*; and *moderately active, nonsmokers and nondrinkers with poor dietary intake* [7]. This highlights the importance of taking a holistic approach to behavioral interventions and targeting multiple health risk behaviors collectively. Transfer theory [9] also suggests that the advantages of addressing multiple health risk behaviors include that the knowledge and experiences acquired to successfully modify one behavior can be applied to change other health behaviors [10]. Given the rates of smoking, nutrition, alcohol consumption, and physical activity risk behaviors among vocational education students, and that such behaviors tend to cluster together, connecting this population to services that provide effective support to modify these health behaviors has the potential to improve health outcomes in this population.

### Effectiveness of Online and Telephone Support Services in Modifying Health Risk Behaviors

Online and telephone interventions are effective in reducing smoking [11,12], improving nutrition [13,14], reducing alcohol consumption [15], increasing physical activity [13,14], and modifying multiple health risk behaviors [13,16], and they provide a means for changing these behaviors among vocational education students. Advantages of telephone and online interventions include privacy and reduction in stigma [17-19], avoidance of travel time and cost [20], potential to reach a large number of people [21,22], ability to reach individuals who may not seek support [23], and capacity to provide support when it is needed most [19].

In the United States and Australia, for example, evidence-based online and telephone interventions designed to modify health risk behaviors are available to the general population at no cost to users [24-26]. Online interventions available for the general population in Australia include QuitCoach (smoking) [27], Tertiary Health Research Intervention Via Email (THRIVE; alcohol) [28], Healthy Eating Quiz (fruit and vegetable consumption), and 10,000 steps (physical activity) programs [29]. Telephone services available to the Australian general population include Quitline (smoking) [30], Alcohol Drug Information Service (ADIS; alcohol), and Get Healthy Information and Coaching Service (GHICS; fruit and vegetable consumption and physical activity) [31]. Despite the efficacy of online and telephone support services in improving health risk behaviors, the uptake of such services is low [32,33]. For example, only 3% to 4% of smokers in Australia and the United States use the Quitline [32]. Less than 1% of adults in New South Wales, Australia, who are overweight and obese call the GHICS to receive support to increase their fruit and vegetable consumption and physical activity [34]. Only 0.3% of smokers reported using an internet-based intervention in an attempt to quit smoking in the past year [35]. Furthermore, when the figures provided in an Australian study were divided by the total Australian population aged 16 years and older [36] with access to the online service, only 0.4% were found to use the Healthy Eating Quiz [37].

The low rates of use of online and telephone support services may partially be due to the passive recruitment approaches (eg, mass media campaigns) used by these services, which generally rely on individuals to initiate contact with the service providers themselves [38]. Alternatively, proactive recruitment involves service providers initiating contact with the individual to offer support services and has been found to increase the uptake of such services [22,39-42]. For instance, 52% of smokers who were proactively offered Quitline telephone support accepted it [30], and 23% of people who were proactively offered the GHICS accepted it [43]. McClure et al [44] reported that 7% of smokers who were proactively invited to use an internet-based intervention (ie, Project Quit) accepted and visited the website and 4% signed up to use the service.

### Factors Associated With Uptake of Support Services

Sociodemographic characteristics are associated with whether people will sign up to use online or telephone interventions. Skov-Ettrup et al [22] found that participants who spent 11 years or more in school compared with those who spent shorter periods in school were less likely to use telephone counseling to quit smoking. Furthermore, participants aged 18 to 39 years were more likely to sign up for an internet-based intervention targeting smoking than those aged 40 years or more [22]. A study conducted in Hong Kong also found that being employed and belonging to a middle-income family was significantly associated with accepting to use a telephone service to quit smoking [45]. Furthermore, Schneider et al [41] found that Dutch adults who were younger, male, highly educated, employed, and were in a relationship were more likely to express interest in the proactively offered online intervention targeting multiple lifestyle behaviors.

Existing studies on the uptake of online and telephone services and factors associated with uptake focus only on the general population or university students [22,45]. Only one study examined the uptake of online support services for multiple health risk behaviors [41], whereas the other studies focused on a single health risk behavior only (eg, smoking) [22,32,44,45]. Despite their high rates of smoking, nutrition, alcohol consumption, and physical activity risk behaviors, it is unknown whether vocational education students will sign up for proactively offered online and/or telephone support to modify one or more of these behaviors. Furthermore, whether the sociodemographic and psychological characteristics of vocational education students are associated with signing up for online and telephone support services is unexplored. Although no previous research has examined whether psychological characteristics are associated with uptake of online and telephone support services targeting multiple health risk behaviors, mental health issues have been found to co-occur or cluster with health risk behaviors [6,46]. Therefore, it is important to examine whether those with symptoms of psychological distress (ie, anxiety and depression) will sign up for support services targeting their health risk behaviors.

### Objectives

The aims of this study are to examine the following among vocational education students in the Technical and Further Education (TAFE) setting:

1. Uptake of proactively offered online and telephone support services for each smoking, nutrition, alcohol consumption, and physical activity risk behavior and multiple health behaviors (ie, at least two behaviors).
2. Sociodemographic and psychological characteristics associated with the uptake of online and telephone support services for each smoking, nutrition, alcohol consumption, and physical activity risk behavior and multiple health behaviors (ie, at least two behaviors).

## Methods

### Study Design and Setting

The data used in this study were gathered from May 2018 to May 2019 as part of a cluster randomized controlled trial examining the effectiveness of electronic feedback and online and telephone support services targeting multiple health risk behaviors among vocational education students. Of the 14 TAFE campuses, 8 campuses were randomized to the intervention group and 6 campuses to the control group. The control campuses received no intervention. The data from all 8 intervention campuses, which were located in the Hunter, Upper Hunter, and Central Coast regions of New South Wales, Australia, are included in this paper. Vocational education students who attended these 8 intervention campuses were proactively offered online and telephone support services for those behaviors where they did not meet Australian health guidelines. The University of Newcastle Human Research Ethics Committee granted ethics approval for this study. Written approval was also provided by the Chief Education and Training Officer from TAFE New South Wales to conduct this research. The trial was registered with the Australian New Zealand Clinical Trials Registry (ACTRN12618000723280).

### Participants

Participants were eligible to participate if they were aged 16 years and above and enrolled in a TAFE class that ran for 6 months or more. Participants were excluded if they met the recommended health guidelines for all smoking, nutrition, alcohol consumption, and physical activity risk behaviors and were not able to read or write English.

### Procedure

The TAFE service coordinator on each campus identified all eligible classes that ran for 6 months or more and approached the head teachers of the department followed by the teachers of the classes to obtain approval to attend those classes. In those classes where the head teacher and teacher granted approval, TAFE students were given an information letter in class about the research 1 week before data collection. On the day of data collection, members of the research team attended the class to administer the online survey. First, a verbal explanation of the study was provided to the class by a researcher, and interested students were given a computer tablet to complete the online questionnaire. Informed consent was obtained online on the first page before the online survey questions. Interested students selected that they would like to participate in the trial before the survey questions appeared on screen.

### Intervention

#### Electronic Feedback

For each smoking, nutrition, alcohol consumption, and physical activity health risk behavior, if a participant did not meet the Australian health guidelines, information appeared on the computer tablet, specifying that they did not meet the recommended guidelines, and information was provided about effective behavior change strategies. Table 1 presents the Australian health guidelines for each risk behavior and the electronic feedback provided to those who did not meet the recommended guidelines for each behavior.

**Table 1:** Australian health guidelines and electronic feedback for each smoking, nutrition, alcohol consumption, and physical activity risk behavior.

Health risk behaviors	Australian health guidelines	Electronic feedback
Smoking tobacco	No smoking of cigarettes or tobacco products [47].	Australian guidelines recommend that not smoking any cigarettes and not using any tobacco improves your health. Quitting smoking can produce immediate and long-term benefits to your health. Quitting smoking can take a few attempts so it is important to keep trying. Strategies that can help you quit include web-based and telephone programs, getting your doctor’s advice and support, and using nicotine replacement therapies. We are offering free help to smokers who would like to stop smoking or would consider stopping in the future.
Nutrition (fruit and vegetable intake)	Eat 2 or more serves of fruit each day and 5 or more serves of vegetables each day [48].	Australian guidelines recommend that adults should eat at least two serves of fruit each day and at least five serves of vegetables each day. Eating healthy can improve your overall health. Strategies that can help you increase your fruit and vegetable intake include web-based and telephone programs, speaking to a dietitian, and getting advice from your doctor. We are offering free help to people who do not eat enough fruit or vegetables and may like assistance to increase the amount of fruit and vegetables they eat.
Alcohol intake	No more than 2 standard alcoholic drinks per day (to reduce life-time disease risk) and no more than 4 standard alcoholic drinks on one occasion (to reduce the risk of injury and acute problems) [49].	Australian guidelines recommend that adults should drink no more than 2 standard drinks of alcohol on one day and no more than 4 standard drinks on one occasion to reduce the risk of alcohol-related harm. Reducing the amount of alcohol you drink can have immediate and long-term benefits to your health. Strategies that can help you reduce your alcohol intake include web-based and telephone programs and getting your doctor’s advice and support. We are offering free help to people who drink more alcohol than recommended and may like assistance to reduce the amount of alcohol they drink.
Physical activity	Do at least 150 to 300 minutes of moderate physical activity or at least 75 to 150 minutes of vigorous physical activity each week [50].	Australian guidelines recommend that adults do 150 to 300 minutes of moderate physical activity or 75 to 150 minutes of vigorous physical activity each week. Increasing your physical activity can improve your overall health. Strategies that can help you increase your physical activity include web-based and telephone programs, regular times to do physical activity, and participating in sports. We are offering free help to people who do not do enough physical activity and may like assistance to increase their physical activity.

#### Proactive Offer of Online and Telephone Services

After the electronic feedback had appeared for each smoking, nutrition, alcohol consumption, and physical activity risk behavior where the participant did not meet Australian health guidelines, both online and telephone services were offered. The online and telephone services were QuitCoach and Quitline for smoking, Healthy Eating Quiz and GHICS for fruit and vegetable consumption, THRIVE and ADIS for risky alcohol consumption, and 10,000 steps and GHICS for physical activity. These support services were chosen because they are existing programs available to the general population in New South Wales for free, and previous studies have demonstrated their effectiveness [27,31,37,51-53]. Participants who agreed to use the online support services were asked to provide their email or mobile phone details. The hyperlinks of the online programs were sent to them. Participants who agreed to sign up for telephone support services were asked to provide their contact details (home and/or mobile phone number) so that the telephone services could contact them.

### Measures

#### Uptake of Proactively Offered Online and Telephone Services

Uptake was measured by participants indicating *yes* they would like to use the specified service and providing the relevant contact details (ie, phone number and/or email address). Uptake referred to signing up to use the services offered.

#### Sociodemographic Characteristics

Age, sex, country of birth, Aboriginal and Torres Strait Islander identity, highest level of education completed, marital status, employment status, and postcode of residence were collected.

#### BMI

Participants were asked to self-report their height in centimeters and weight in kilograms. Conversion tables were displayed in the questionnaire to help participants if they required assistance converting their height from feet and inches into centimeters or their weight from stone into kilograms. Participants’ BMI scores were calculated and categorized as underweight (BMI<18.5 kg/m^2^), healthy weight (BMI 18.5-24.9 kg/m^2^), overweight (BMI 25-29.9 kg/m^2^), and obese (BMI≥30 kg/m^2^).

#### Anxiety and Depression

The Patient Health Questionnaire-4 (PHQ-4) is a reliable and valid measure that was used to measure anxiety and depression [54]. The PHQ-4 asks, *Over the last 2 weeks, how often have you been bothered by the following problems?* The 2 questions used to measure depression were (1) *Little interest or pleasure in doing things* and (2) *Feeling down, depressed or hopeless*. The 2 items used to measure anxiety were (1) *Feeling nervous, anxious or on edge* and (2) *Not being able to stop or control worrying*. Response options were as follows: not at all (0), several days (1), more than half the days (2), and nearly every day (3) [54]. Scores on both the anxiety and depression scale were dichotomized into yes (ie, those with a total score ≥3 for anxiety or depression) and no (ie, those with a total score <3 for anxiety or depression).

#### Intention to Change

Participants’ intentions to change the behaviors for which they did not meet Australian guidelines were assessed by asking what best described their intentions regarding (1) quitting smoking, (2) reducing alcohol consumption, (3) increasing daily fruit intake, (4) increasing daily vegetable intake, and (5) increasing weekly physical activity. The response options were as follows: will quit (for smoking) or reduce (for alcohol) or increase (for fruit, vegetables, and physical activity) in the next 30 days; will quit (for smoking) or reduce (for alcohol) or increase (for fruit, vegetables, and physical activity) in the next 6 months; may quit (for smoking) or reduce (for alcohol) or increase (for fruit, vegetables, and physical activity) in the future, but not in the next 6 months; never expect to quit (for smoking) or reduce (for alcohol) or increase (for fruit, vegetables, and physical activity); and do not know.

### Statistical Analysis

All analyses were performed using SAS version 9.3 (SAS Institute Inc). Frequencies and percentages were used to describe categorical data (ie, sociodemographic characteristics, BMI, and psychological characteristics). Mean and standard deviation were used to describe the continuous data (ie, age of respondents) in the demographics table. The uptake of proactively offered online and telephone support services was described using frequencies and percentages. Four multiple logistic regression models were used to examine whether sociodemographic characteristics, psychological characteristics, and intention to change were associated with signing up for telephone or online services for each smoking, nutrition, alcohol consumption, and physical activity risk behavior. BMI was also included in the regression models for nutrition and physical activity that examined the characteristics associated with signing up for telephone or online support services for these behaviors. A fifth logistic regression model was used to identify whether sociodemographic factors, psychological characteristics, and intention to change were associated with signing up for telephone or online support services for multiple health risk behaviors (ie, at least two risky behaviors).

## Results

### Participants’ Characteristics

Overall, 551 participants had at least one health risk behavior and were offered both online and telephone support services. Table 2 shows the sociodemographic characteristics, BMI, and psychological characteristics of the sample.

**Table 2:** Sociodemographic, BMI, psychological characteristics, and vocational education courses of the sample (N=551).

Characteristic		Value
**Gender, n (%)**		
	Male	310 (56.3)
	Female	228 (41.4)
	Other	13 (2.4)
Age, years, mean (SD)		25.7 (11.1)
**Country of birth, n (%)**		
	Australia	509 (92.4)
	Other	42 (7.6)
**Highest level of education completed, n (%)**		
	High school or less	388 (70.4)
	TAFE^a^ or university	163 (29.6)
**Marital status, n (%)**		
	Never married	345 (62.6)
	Married or living with partner	166 (30.1)
	Divorced or separated or widowed	40 (7.3)
**Employment status, n (%)**		
	Employed	432 (78.4)
	Unemployed	119 (21.6)
**Aboriginal and Torres Strait Islander, n (%)**		
	Yes	72 (13.1)
	No	479 (86.9)
**Residence** ^b^ **, n (%)**		
	Urban	331 (64.4)
	Rural	183 (35.6)
**BMI status** ^c^ **, n (%)**		
	Underweight	21 (4.0)
	Healthy weight	241 (45.6)
	Overweight	147 (27.8)
	Obese	119 (22.5)
**Depression, n (%)**		
	Yes	131 (23.8)
	No	420 (76.2)
**Anxiety, n (%)**		
	Yes	163 (29.6)
	No	388 (70.4)
**Vocational education course, n (%)**		
	Automotive mechanic	165 (29.9)
	Community service or mental health	56 (10.2)
	Floristry	49 (8.9)
	Fabrication and welding	38 (6.9)
	Hairdressing	37 (6.7)
	Mechanical engineering	34 (6.9)
	Animal studies or veterinary nursing	33 (6.0)
	Arts	23 (4.2)
	Dental nursing	22 (4.0)
	Electrotechnology	21 (3.8)
	Protective coating	19 (3.5)
	Programming	15 (2.7)
	Baking	13 (2.4)
	Mobile plant technology	12 (2.2)
	Photography	7 (1.2)
	Commercial cookery	5 (0.9)
	Business administration	2 (0.4)

^a^TAFE: Technical and Further Education.

^b^Data missing for residence, n=37.

^c^Data missing for BMI, n=23.

### Uptake of Proactively Offered Online and Telephone Support Services

Table 3 outlines the uptake of online and telephone support services for each smoking, nutrition, alcohol consumption, and physical activity risk behavior as well as for multiple health risk behaviors. Among those who smoked tobacco, 6.8% (13/191) and 2.6% (5/191) signed up to use QuitCoach and Quitline, respectively, to help them quit smoking, whereas 6.8% (13/191) signed up to use either Quitline or QuitCoach. For participants not meeting the recommended guidelines for fruit and vegetable intake, 14.3% (58/406) and 3.9% (16/406) signed up to use the Healthy Eating Quiz and GHICS, respectively, to improve their fruit and vegetable intake. A total of 14.5% (59/406) signed up for either the Healthy Eating Quiz or GHICS. Of those who were offered online and telephone services to reduce their alcohol consumption, 5.5% (18/327) signed up for THRIVE and 0.9% (3/327) signed up for ADIS, with 5.5% (18/327) signing up for either ADIS or THRIVE. Among those who did not meet the physical activity guidelines, 11.4% (26/228) signed up for 10,000 steps and 7.0% (16/228) signed up for GHICS to improve their physical activity; 12.7% (29/228) signed up for either 10,000 steps or GHICS to modify their physical activity.

**Table 3:** Uptake of proactively offered online and telephone support services (N=551).

Health risk behaviors and online and telephone interventions		Eligible students offered the support services, n		Signed up to support services, n (%)	
**Smoking**					
	Quitline (telephone)		191		5 (2.6)
	QuitCoach (online)		191		13 (6.8)
	Quitline or QuitCoach		191		13 (6.8)
**Inadequate fruit and vegetable consumption**					
	Get Healthy Information and Coaching Service (telephone)		406		16 (3.9)
	Healthy Eating Quiz (online)		406		58 (14.3)
	Healthy Eating Quiz or Get Healthy Information and Coaching Service		406		59 (14.5)
**Risky alcohol consumption**					
	ADIS^a^ (telephone)		327		3 (0.9)
	THRIVE^b^ (online)		327		18 (5.5)
	THRIVE or ADIS		327		18 (5.5)
**Physical inactivity**					
	Get Healthy Information and Coaching Service (telephone)		228		16 (7.0)
	10,000 steps (online)		228		26 (11.4)
	10,000 steps or Get Healthy Information and Coaching Service		228		29 (12.7)
**Uptake of support services for multiple behaviors**					
	Uptake of telephone services for multiple behaviors (eg, GHICS^c^ and ADIS)		377		8 (2.1)
	Uptake of online services for multiple behaviors (eg, THRIVE and Healthy Eating Quiz)		377		21 (5.6)
	Uptake of any service (online or telephone) for at least two health behaviors (eg, THRIVE for alcohol and GHICS for physical activity)		377		22 (5.8)

^a^ADIS: Alcohol Drug Information Service.

^b^THRIVE: Tertiary Health Research Intervention Via Email.

^c^GHICS: Get Healthy Information and Coaching Service.

Of the participants who engaged in multiple health risk behaviors, 5.6% (21/377) signed up for online support services for multiple behaviors and 2.1% (8/377) signed up for telephone services for multiple behaviors. For participants with multiple health risk behaviors, 5.8% (22/377) signed up for either telephone or online services for at least two health behaviors.

### Characteristics Associated With the Uptake of Proactively Offered Telephone or Online Support Services for Health Risk Behaviors

The characteristics associated with the uptake of proactively offered telephone or online support services for smoking and alcohol consumption (Table 4), nutrition and physical activity (Table 5), and at least two health risk behaviors (Table 6) have been presented.

**Table 4:** Characteristics associated with uptake of telephone or online support services for smoking and alcohol consumption.

Characteristics		Uptake of QuitCoach or Quitline (smoking)			Uptake of THRIVE^a^ or ADIS^b^ (alcohol)				
		n (%)	Odds ratio (95% CI)	*P* value		n (%)	Odds ratio (95% CI)		*P* value
**Gender**				.30				.17	
	Male	8 (6.4)	Ref^c^			13 (6.2)	Ref		
	Female	5 (8.3)	0.33 (0.04-2.65)			5 (4.6)	0.38 (0.10-1.51)		
**Age (years)**				.64				.49	
	16-39	11 (6.4)	0.55 (0.04-6.93)			15 (5.2)	0.50 (0.07-3.63)		
	>40	2 (18.2)	Ref			3 (13.6)	Ref		.
**Highest level of education completed**				.72				.63	
	High school or less	9 (5.8)	0.72 (0.12-4.20)			14 (5.5)	1.41 (0.34-5.80)		
	University or TAFE^d^	4 (10.8)	Ref			4 (5.7)	Ref		
**Marital status**				.77				.42	
	Never married	8 (6.1)	0.70 (0.14-3.56)			9 (4.1)	0.64 (0.21-1.97)		
	Divorced or separated or widowed	2 (13.3)	1.75 (0.13-23.00)			3(15.0)	2.14 (0.32-14.58)		
	Married or living with partner	3 (6.7)	Ref			6 (7.0)	Ref		
**Employment**				.02				.68	
	Employed	8 (4.8)	0.10 (0.01-0.72)			14 (5.0)	0.72 (0.15-3.37)		
	Unemployed	5 (20.0)	Ref			4 (8.2)	Ref		
**Residence**				.07				.86	
	Urban	6 (5.8)	0.23 (0.05-1.15)			12 (6.4)	1.10 (0.37-3.29)		
	Rural	7 (9.7)	Ref			6 (5.1)	Ref		
**Depression**				.03				.57	
	Yes	2 (3.3)	Ref			3 (3.8)	Ref		
	No	11(8.4)	10.25 (1.30-80.67)			15 (6.1)	1.53 (0.35-6.75)		
**Anxiety**				.02				.43	
	Yes	6 (9.0)	Ref			5 (4.9)	Ref		
	No	7 (5.7)	0.11 (0.02-0.71)			13 (5.8)	0.59 (0.16-2.22)		
**Aboriginal or Torres Strait Islander**				.28				.34	
	Yes	2 (5.6)	0.34 (0.05-2.39)			1 (2.4)	0.35 (0.04-3.05)		
	No	11 (7.1)	Ref			17 (6.0)	Ref		
**Intention to change**				.08				.13	
	No intention to change in 6 months or did not know	5 (3.7)	Ref			12 (4.3)	Ref		
	Intention to change in 30 days	4 (16.0)	5.46 (1.11-26.97)			4 (13.8)	3.01 (0.76-11.95)		
	Intention to change in 6 months	4 (12.5)	3.96 (0.77-20.27)			2 (11.8)	3.63 (0.67-19.62)		

^a^THRIVE: Tertiary Health Research Intervention Via Email.

^b^ADIS: Alcohol and Drug Information Services.

^c^Ref: Reference category

^d^TAFE: Technical and Further Education.

**Table 5:** Characteristics associated with the uptake of telephone or online support services for nutrition and physical inactivity.

Characteristics		Uptake of Healthy Eating Quiz or GHICS^a^ (fruit and vegetables)			Uptake of 10,000 steps or GHICS (physical activity)		
		n (%)	Odds ratio (95% CI)	*P* value	n (%)	Odds ratio (95% CI)	*P* value
**Gender**				.60			.34
	Male	25 (10.9)	Ref^b^		6 (6.1)	Ref	
	Female	33 (20.0)	1.22 (0.58-2.56)		22 (17.7)	1.80 (0.54-6.05)	
**Age (years)**				.78			.72
	16-39	48 (14.1)	1.15 (0.42-3.15)		18 (10.0)	0.78 (0.20-3.02)	
	>40	11 (22.5)	Ref		11 (25.6)	Ref	
**Highest level of education completed**				.42			.76
	High school or less	34 (11.9)	0.75 (0.38-1.50)		13 (9.1)	0.85 (0.30-2.39)	
	University or TAFE^c^	25 (20.7)	Ref		16 (18.8)	Ref	
**Marital status**				.65			.56
	Never married	31 (12.3)	0.79 (0.37-1.67)		13 (10.2)	1.00 (0.31-3.22)	
	Divorced or separated or widowed	4 (11.8)	0.60 (0.17-2.11)		5 (25.0)	2.31 (0.49-10.90)	
	Married or living with partner	24 (20.0)	Ref		11 (13.7)	Ref	
**Employment**				.07			.11
	Employed	33 (10.5)	0.48 (0.22-1.05)		10 (6.5)	0.42 (0.14-1.24)	
	Unemployed	26 (28.6)	Ref		19 (25.3)	Ref	
**Residence**				.17			.65
	Urban	45 (18.5)	1.65 (0.81-3.38)		21 (14.3)	1.29 (0.44-3.80)	
	Rural	13 (9.6)	Ref	.	7 (10.3)	Ref	
**Depression**				.20			.36
	Yes	18 (17.7)	Ref		10 (15.9)	Ref	
	No	41 (13.5)	0.58 (0.26-1.32)		19 (11.5)	0.57 (0.17-1.90)	
**Anxiety**				.29			.90
	Yes	19 (16.2)	Ref		13 (16.3)	Ref	
	No	40 (13.8)	1.58 (0.68-3.64)		16 (10.8)	1.08 (0.32-3.62)	
**Aboriginal or Torres Strait Islander**				.19			.29
	Yes	5 (10.0)	0.43 (0.12-1.52)		3 (10.0)	0.41 (0.08-2.13)	
	No	54 (15.2)	Ref		26 (13.1)	Ref	
**BMI status**				.23			.13
	Overweight	11 (10.5)	0.60 (0.25-1.42)		9 (16.1)	3.43 (1.01-11.65)	
	Obesity	20 (23.3)	1.39 (0.65-2.97)		12 (21.1)	2.34 (0.68-8.01)	
	Healthy weight or underweight	26 (13.2)	Ref		7 (7.1)	Ref	
**Intention to change**				.25			.04
	No intention to change in 6 months or did not know	25 (11.5)	Ref		7 (6.4)	Ref	
	Intention to change in 30 days	23 (20.2)	1.76 (0.87-3.56)		15 (24.2)	4.01 (1.33-12.07)	
	Intention to change in 6 months	10 (14.1)	1.57 (0.67-3.66)		7 (12.3)	1.57 (0.42-5.86)	

^a^GHICS: Get Healthy Information and Coaching Service.

^b^Ref: Reference category

^c^TAFE: Technical and Further Education.

**Table 6:** Characteristics associated with the uptake of telephone or online support services for multiple health risk behaviors.

Characteristics		Uptake of any service for multiple health risk behaviors		
		n (%)	Odds ratio (95% CI)	*P* value
**Gender**				.74
	Male	7 (3.2)	Ref^a^	
	Female	14 (9.4)	1.22 (0.38-3.86)	
**Age (years)**				.75
	16-39	16 (5.0)	1.25 (0.32-4.94)	
	>40	6 (15.4)	Ref	
**Highest level of education completed**				.69
	High school or less	12 (4.4)	0.81 (0.29-2.27)	
	University or TAFE^b^	10 (9.9)	Ref	
**Marital status**				.24
	Never married	9 (3.9)	0.47 (0.15-1.45)	
	Divorced or separated or widowed	4 (13.3)	1.69 (0.37-7.69)	
	Married or living with partner	9 (8.0)	Ref	
**Employment**				.003
	Employed	8 (2.7)	0.18 (0.06-0.56)	
	Unemployed	14 (18.2)	Ref	
**Residence**				.85
	Urban	16 (7.2)	0.89 (0.29-2.79)	
	Rural	6 (4.7)	Ref	
**Depression**				.13
	Yes	10 (10.2)	Ref	
	No	12 (4.3)	0.41 (0.13-1.30)	
**Anxiety**				.94
	Yes	10 (8.5)	Ref	
	No	12 (4.6)	1.04 (0.31-3.50)	
**Aboriginal or Torres Strait Islander**				.54
	Yes	3 (5.7)	0.60 (0.12-2.98)	
	No	19 (5.9)	Ref	
**Intention to change at least two behaviors**				.49
	No intention to change at least two behaviors	14 (5.1)	Ref	
	Intention to change at least two behaviors	8 (8.0)	1.43 (0.52-3.93)	

^a^Ref: Reference category.

^b^TAFE: Technical and Further Education.

### Characteristics Associated With Signing Up for QuitCoach or Quitline for Smoking and for THRIVE or ADIS for Alcohol Consumption

After adjusting for the covariates, vocational education students who reported not being depressed had greater odds (odds ratio [OR] 10.25, 95% CI 1.30-80.67) of signing up for QuitCoach or Quitline than those who were depressed. Those who were employed (OR 0.10, 95% CI 0.01-0.72) or reported not being anxious (OR 0.11, 95% CI 0.02-0.71) had smaller odds of signing up for QuitCoach or Quitline.

None of the characteristics were associated with signing up for THRIVE or ADIS to modify alcohol consumption after adjusting for the covariates.

### Characteristics Associated With Signing Up for Healthy Eating Quiz or GHICS for Fruit and Vegetable Intake and for 10,000 Steps or GHICS for Physical Activity

After adjusting for the covariates, none of the factors were associated with signing up for the Healthy Eating Quiz or GHICS for fruit and vegetable consumption.

After adjusting for the covariates, vocational education students who intended to increase their physical activity within 30 days had greater odds (OR 4.01, 95% CI 1.33-12.07) of signing up for 10,000 steps or the GHICS than those who did not intend to change in 6 months or did not know if they intended to change.

### Characteristics Associated With Signing Up for Services for Multiple Health Risk Behaviors

Among vocational education students who engaged in multiple health risk behaviors, those who were employed had significantly smaller odds (OR 0.18, 95% CI 0.06-0.56) of signing up for support services for at least two behaviors than those who were unemployed after adjusting for the covariates.

## Discussion

### Principal Findings

This study examined the uptake of proactively offered online and telephone support services targeting smoking, nutrition, alcohol consumption, and physical activity risk behaviors and multiple health risk behaviors among vocational education students. More than half of the participants were men. Given that most health behavior studies have an overrepresentation of women, this study, in contrast, presents findings where men are well represented and can be reached via TAFE [55]. The findings revealed that the uptake of online and telephone services that targeted smoking, inadequate fruit and vegetable consumption, risky alcohol consumption, and physical inactivity was low among vocational education students. We also found that less than 10% of vocational education students who engaged in multiple health risk behaviors signed up for online or telephone support services to modify at least two health risk behaviors. Vocational education students who were employed and those who did not have symptoms of anxiety were less likely to sign up for support services targeting smoking. Vocational education students who reported no symptoms of depression were more likely to sign up for support services targeting smoking. Vocational education students who intended to change their physical activity in the next 30 days were more likely to sign up for physical activity support services than those who did not intend to change in 6 months or did not know. Finally, vocational education students who were employed were less likely to sign up for support services for at least two health risk behaviors.

### Comparison With Prior Work

Uptake of proactively offered telephone support for smoking was relatively low in our study (2.6%) compared with an Australian study that proactively offered telephone support to smokers in the general population (52%) [30] and compared with 82.9% in a study conducted in Hong Kong [45] and 74% in a Denmark study [22]. Differences in the characteristics of the populations in these 3 studies compared with the younger group in this study may provide some explanation for the lower uptake of proactively offered telephone support in vocational education students than in the general population. For example, younger adults may feel that they will not experience health consequences of smoking in the near future and, consequently, may be less likely to wish to quit smoking than their older counterparts. Furthermore, women may be more likely to sign up for support services than men [22,56], and this may in part account for the higher uptake of smoking telephone services reported in previous studies [22,30,45]. Uptake of proactively offered online support (QuitCoach) targeting smoking in our study (6.8%) was similar to that reported by McClure et al [44] in their study (7%). The study by Skov-Ettrup et al [22] reported a higher uptake (69%) of internet intervention for smoking compared with our study. Although the uptake of telephone support for smoking cessation was low (2.6%) among vocational education students, it is comparable with the proportion of smokers in the general population that use the Quitline in Australia (4%) [32] and in the United States (3.5%) [33]. There were no direct age or socioeconomic status comparable data on uptake in these 2 studies. There are a number of potential reasons for the low uptake of smoking services among vocational education students. First, vocational education students may not wish to quit smoking and may believe there are advantages to smoking that override any negative consequences. Second, they may perceive that they are not addicted to cigarettes and feel that they could quit smoking unassisted if they wanted to. Third, they may be part of a social network where their peers smoke tobacco and may feel that they need to continue to smoke to feel socially accepted by their peers. Additional reasons may include their lack of readiness to change their health risk behaviors, perceived inappropriateness of the services they were offered, their preference to change by themselves, and a belief that the support services they were offered would not help them [57].

Compared with the less than 1% of adults in New South Wales who are overweight and obese and who reported using the GHICS [34], our study achieved higher rates (7.0% for physical inactivity and 3.9% for fruit and vegetables) of signing up for this service. However, the rates of vocational education students signing up for this service were lower compared with what was reported in the study by Wolfenden et al [58], in which 23% of New South Wales residents aged 18 years and above in the general community agreed to allow their details to be forwarded to the GHICS when they were proactively called. Furthermore, our study reported a higher (14.3%) rate of signing up for the Healthy Eating Quiz for fruit and vegetables compared with the study where only 0.4% of people aged 16 years and above signed up to use the Healthy Eating Quiz [37]. There is no existing research that has examined the uptake of proactively offered online and telephone support services targeting risky alcohol consumption; therefore, no comparisons can be made with this study.

Although the uptake of support services was low across the health risk behaviors, vocational education students appeared to prefer online support services (5.5%-14.3%) to telephone services (0.9%-7.0%) targeting each behavior and multiple health risk behaviors. This is not surprising given that the TAFE setting is mostly composed of young adults, who are more likely to use the internet [59] and prefer online support services [60]. The convenience of being able to access online programs when they wished to rather than scheduling a particular time to speak with an advisor may also explain why vocational education students preferred online programs to telephone services. In addition, in vocational education students, the uptake of any support services targeting nutrition and physical activity (12.7%-14.5%) appeared higher than that of services for smoking and alcohol (5.5%-6.8%). Possible explanations include that for physical activity and fruit and vegetable consumption, the goal is to increase healthy behaviors, whereas changes to smoking and alcohol consumption involve stopping risky behaviors. Vocational education students may smoke tobacco or drink alcohol to help them cope with stress, depression, or anxiety [61], during social gatherings including work functions with colleagues [62,63], or to feel socially accepted and to improve self-confidence [62,63]. As a result, vocational education students may be less likely to sign up for services to change smoking and alcohol behaviors than nutrition and physical activity behaviors.

Those who were employed had smaller odds of signing up for support services (ie, Quitline or QuitCoach) for smoking and for services for at least two health risk behaviors. This is in contrast with the study by Mak et al [45], which examined the factors that influenced parents who smoke to participate in a proactive telephone intervention for smoking, and the study by Schneider et al [41], which reported that participants who were employed were more likely to participate in an online intervention targeting multiple lifestyle behaviors [41]. Students who worked may have felt that they had less time compared with students who did not work and did not want to sign up for support services they could not commit the time to. We also found that participants who reported not being depressed were more likely to sign up for support services that targeted smoking, whereas those who were not anxious were less likely to sign up for online or telephone support services for smoking. Previous research has found a relationship between smoking cessation and improved mental health [64]. Vocational education students who are smokers and experiencing symptoms of anxiety may thus feel more compelled to sign up for smoking support services not only to quit smoking but also to improve their mental health [64]. Smoking cessation interventions should therefore not only focus on the physical addiction of smoking but also consider the psychological needs of service users to offer coping strategies that may be useful not only for quitting smoking but also for reducing anxiety and psychological distress. Vocational education students who intended to increase their physical activity in 30 days were more likely to sign up for 10,000 steps or the GHICS compared with those who did not intend to increase their physical activity in the next 6 months or did not know if they would change. Intention to change was associated with the uptake of physical activity services but not with the uptake of smoking, nutrition, and alcohol support services. These results suggest that smoking, nutrition, and alcohol support services should be offered to all vocational education students who do not meet the recommended guidelines, irrespective of their intention to change. Physical activity services may benefit from targeting their messaging and recruitment efforts to those intending to increase their physical activity in 30 days. Interventions that target health risk behaviors should therefore consider how to promote their services to vocational education students who do not meet the recommended guidelines for each behavior and how to incorporate strategies to motivate vocational education students to sign up for support services.

Our study offered existing online and telephone services to vocational education students. Although some services such as the GHICS targeted multiple behaviors (ie, fruit and vegetable intake and physical activity), others such as the Quitline focused on one behavior (ie, smoking). There is no telephone service in Australia that addresses all smoking, nutrition, alcohol consumption, and physical activity risk behaviors collectively. Given that smoking, nutrition, alcohol consumption, and physical activity risk behaviors cluster together in vocational education students [6-8] and that transfer theory [10] suggests that modifying one health risk behavior can lead to changes in other behaviors, future studies may wish to offer vocational education students interventions in a form where all smoking, nutrition, alcohol consumption, and physical activity risk behaviors can be addressed by the same service simultaneously.

### Limitations

This study had some limitations. First, to be eligible to participate in this study, which was part of a cluster randomized controlled trial, vocational education students needed to be enrolled in a class that ran for at least six months. Therefore, these findings may not be generalizable to vocational education students enrolled in courses that run for less than 6 months. Second, vocational education classes were recruited from the Hunter, Upper Hunter, and Central Coast areas of New South Wales and may not be representative of all vocational education campuses across Australia or internationally.

### Conclusions

Although most vocational education students who were offered online and telephone support services did not sign up for these, the uptake rates for some of the support services appear to be higher than self-initiated uptake in the general population. Scaling up the proactive offer of online and telephone services may produce beneficial health outcomes. Proactively offering support services to vocational education students is sustainable via a system whereby students receive electronic feedback about health risk behaviors and referral to existing online and telephone services as part of the standard vocational education enrollment procedure and via student services. The use of existing online and telephone services is an important strength that supports the sustainability of such an intervention in a vocational education setting.

The findings from this study also suggest that vocational education students prefer online support services to telephone services that target smoking, nutrition, alcohol consumption, physical activity and multiple health risk behaviors. Uptake of support services for nutrition and physical activity also appeared higher than that for smoking and alcohol health risk behaviors. This provides important information for developing health interventions for vocational education students in terms of the mode of delivering interventions to vocational education students and the behaviors they prioritize to change. Future studies should also explore what vocational education students perceive to be the barriers that hinder their uptake of online and telephone support services targeting multiple health risk behaviors and what strategies they would be more likely to use.

## Abbreviations

ADIS: Alcohol and Drug Information Services
GHICS: Get Healthy Information and Coaching Service
NHMRC: National Health and Medical Research Council
OR: odds ratio
PHQ-4: Patient Health Questionnaire-4
TAFE: technical and further education
THRIVE: Tertiary Health Research Intervention Via Email
